# Comparison of the effects of low- versus high-supervision exercise on breast cancer survivorship outcomes

**DOI:** 10.1093/jncics/pkag004

**Published:** 2026-01-25

**Authors:** Kira Bloomquist, Rosalind R Spence, Dimitrios Vagenas, Christopher Pyke, Carolina X Sandler, Sheree Rye, Leonie Young, Sandra C Hayes

**Affiliations:** Center for Health Research (UCSF), Copenhagen University Hospital, Rigshospitalet, Denmark; Viertel Cancer Research Centre, Cancer Council Queensland, Brisbane, QLD, Australia; School of Exercise and Nutrition Sciences, Faculty of Health, Queensland University of Technology, Brisbane, QLD, Australia; School of Public Health and Social Work, Queensland University of Technology, Brisbane, QLD, Australia; Mater Health Services, Brisbane, QLD, Australia; Mater Clinic Unit Faculty of Medicine, The University of Queensland, Brisbane, QLD, Australia; School of Health Sciences, University of New South Wales, Sydney, NSW, Australia; Viertel Cancer Research Centre, Cancer Council Queensland, Brisbane, QLD, Australia; The Wesley Hospital Choices Cancer Support Centre, Brisbane, QLD, Australia; Viertel Cancer Research Centre, Cancer Council Queensland, Brisbane, QLD, Australia; Centre for Health Services Research, The University of Queensland, Brisbane, QLD, Australia

## Abstract

**Background:**

Supervised exercise may provide greater functional and quality of life benefits than unsupervised programs after cancer and is recommended for those with or at risk of breast cancer-related lymphedema. These exploratory analyses compared the effect of low- vs high-supervision exercise on the secondary survivorship outcomes of the SAFE breast cancer trial.

**Methods:**

This randomized study (ANZCTR: ACTRN12616000547448) compared a 12-week exercise program (target 150 min.week^−1^, moderate intensity) supported by either 5 (low supervision [LOW]) or 20 (high supervision [HIGH)] supervised sessions. Inclusion criteria included: stage II+ breast cancer within 5 years, ≥1 comorbidity and/or treatment-related adverse effect, and insufficiently active. Outcomes included lymphedema (self-report and bioimpedance spectroscopy), arm symptoms, upper-extremity function (Patient Reported Outcomes Measurement Information System [PROMIS] Bank v1.2-Upper-Extremity), fatigue, pain, pain interference, pain intensity, physical function, sleep disturbance, anxiety, depression, and satisfaction with social roles (PROMIS-43 Profile v1.0). Chi-square tests evaluated between-group symptom changes. Generalized estimating equations assessed time, group, and time×group effects under an intention-to-treat, 2-sided framework.

**Results:**

Sixty women (mean age, 50 years) were randomized to LOW (*n* = 30) vs HIGH (*n* = 30). At follow-up, both groups showed similar lymphedema prevalence, comparable rates of maintained or improved arm symptoms, and within-group improvements (*P* < .05) in fatigue, physical function, sleep, anxiety, depression, and satisfaction with social roles and activities. Potential for superior benefit in HIGH vs LOW was observed for self-reported range of movement, upper-extremity function, and pain interference and intensity (*P* < .05).

**Conclusion:**

Findings indicate that breast cancer survivors with or at risk of lymphedema can benefit from exercise, even when supervision is limited.

## Graphical abstract

**Figure:**
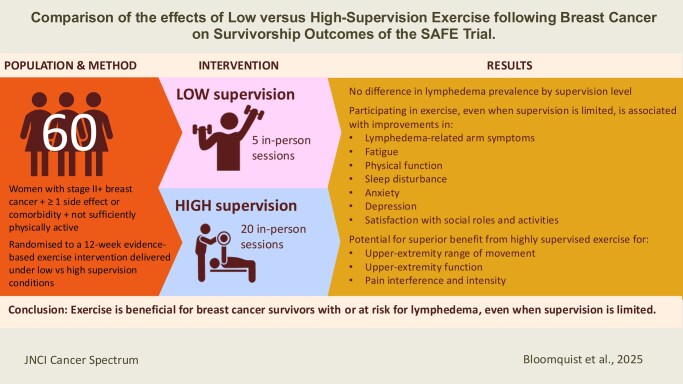

## Introduction

Breast cancer is the second most common cancer worldwide, accounting for 2.26 million new cases in 2020.^1^ With rising incidence and improved survival,^2^ more people are living with treatment sequelae such as fatigue, psychological distress, musculoskeletal issues, and lymphedema, which adversely affect physical and upper-extremity function.^3-5^

Breast cancer-related lymphedema (BCRL) is pathological swelling of the affected arm, hand, breast, or trunk, though the arm is the most commonly measured site. It is associated with symptoms such as pain, heaviness, and tightness,^6^^,^^7^ and affects approximately 20% of breast cancer survivors,^8^ impairing multiple aspects of daily living.^9-11^ Although its exact pathophysiology remains unclear,^12^^,^^13^ treatments including chemotherapy, radiotherapy, and axillary lymph node dissection are consistently associated with elevated risk.^8^ Evidence also supports modifiable risk factors including body mass index (BMI) ≥25 kg.m^−2^ and insufficient physical activity levels,^8^^,^^14^^,^^15^ lending opportunity for mitigation.

Exercise, a structured form of physical activity,^16^ has been studied in clinical trials to assess effect on lymphedema risk and burden.^17-19^ A systematic review and meta-analysis undertaken to summarize the impact of exercise on lymphedema found that exercise did not increase lymphedema risk (*n* = 1955, 12 studies).^18^ Findings also indicated that while exercise did not lead to reduced swelling for those with lymphedema (*n* = 1741, 36 studies), improvements (*P* < .05) in other outcomes including fatigue, pain, and upper-extremity function were observed.^18^ As such, overall findings found exercise to be safe regarding lymphedema, and with the potential for improvement in breast cancer-relevant health outcomes.

Informing these review findings were studies using varied supervision levels, with subgroup analyses indicating similar results irrespective of supervision level.^18^ In contrast, 2019 guidelines from the American College of Sports Medicine state that while supervised resistance exercise was considered safe regarding BCRL, evidence was insufficient to draw similar conclusions for unsupervised or aerobic exercise.^19^ These guidelines mark progress in exercise and lymphedema understanding, particularly in light of historical concerns that exercise might increase risk or exacerbate existing BCRL,^19^^,^^20^ but recommendations may unnecessarily discourage those without access to supervision from participating.

Beyond lymphedema, meta-analyses and individual patient meta-analyses suggest exercise is more effective at improving physical function, quality of life, muscle strength, aerobic fitness, anxiety, and depression when supervised vs unsupervised.^19^^,^^21-23^ However, head-to-head comparisons within 1 study are limited, warranting further study to improve specificity of exercise recommendations. To address these knowledge gaps, this study compared a 12-week exercise intervention delivered with 5 vs 20 supervised in-person sessions. This article reports results on BCRL and associated arm symptoms, upper-extremity function, fatigue, pain interference and intensity, physical function, sleep disturbance, anxiety, depression, and satisfaction with social roles and activities—exploratory outcomes collected as part of the SAFE trial.^24^

## Methods

The SAFE trial was a parallel group, randomized trial which has been described elsewhere.^24^^,^^25^ Ethical approval was obtained from Human Research Ethics Committees at the Queensland University of Technology and participating hospitals, following the Helsinki Declaration. People with lived experience of breast cancer were actively involved in all stages of this study—design, conduct, article preparation, and dissemination. The trial was registered at the Australian New Zealand Clinical Trial Registry (ACTRN12616000547448, April 2016; www.anzctr.org.au) and reported per Consolidating Standards of Reporting Clinical Trials guidelines.^26^

### Participants

Participants were recruited from 3 hospitals in Brisbane, Australia, advertisements or the clinical trials registry. Women were screened via telephone for eligibility: ≥18 years of age, Brisbane resident, undergoing or within 5 years of stage II+ breast cancer treatment, <150 min week^−1^ structured exercise, and presence of at least 1 comorbidity or chronic disease (eg, hypertension, arthritis, obesity, osteoporosis, type II diabetes) OR at least moderate-intensity side effect related to breast cancer treatment (eg, neuropathy, fatigue).

### Randomization

Following written consent and baseline assessment, participants were allocated (computer-generated blocks of 4, stratified by treatment status) to low supervision (LOW) or high supervision (HIGH). Allocations were sealed in sequentially numbered opaque envelopes and blinded to staff until baseline assessment completion.

### Interventions

Both groups participated in a 12-week, home-based, progressive program targeting 150 min per week of moderate-intensity aerobic and resistance exercise. Three accredited exercise physiologists, with study-specific training, delivered the interventions. Exercise type, intensity, and duration were individualized by interests, goals, fitness, side effects, and comorbidities. Using a patient-centered approach,^27^ sessions addressed exercise prescription, technique, intensity, and behavior change support. Intensity was monitored using rating of perceived exertion (6-20).^28^ Participants also received an exercise information workbook and logbook. Adverse events were collected systematically throughout the intervention.^24^ As part of this process, participants were encouraged to report signs or symptoms of new or worsening BCRL, triggering referral to a lymphedema therapist, and individual exercise modifications. Groups differed only in the number of supervised sessions.

#### Low supervision

*Low supervision* participants received 5 supervised, in-person exercise sessions. This is the maximum number of government-funded exercise sessions per year in Australia for people who have a Chronic Disease Management Plan.^29^ The first supervised session was scheduled during week 1, with the remaining supervised sessions scheduled per participant needs.

#### High supervision

*High supervision* participants received 20 supervised, in-person sessions. Two sessions per week were scheduled in weeks 1-8, then 1 supervised session per week in weeks 9-12.

### Outcomes of interest

Outcomes were collected by staff, blinded to group allocation, at baseline and after the 12-week intervention. Self-reported data were obtained via participant-administered questionnaires.

#### Breast cancer-related lymphedema

Objectively measured: Extracellular fluid was assessed using bioimpedance spectroscopy (SFB7, Impedimed, Brisbane, Australia; Supplementary Methods 1).^30-33^ Self-reported lymphedema diagnosis: Participants were asked, “Since diagnosis of your breast cancer, have you been diagnosed with lymphedema (swelling) in the arm, breast or trunk.” If “yes,” participants were instructed to specify the affected area, when they were diagnosed, and to characterize their lymphedema type (single episode, recurrent, persistent), severity (if they had been told that lymphedema was mild, moderate, or severe), stage (ISL staging system: stages 0-III),^7^ pitting status (pitting or nonpitting), and whether they currently had lymphedema (yes/no).

#### Self-reported arm symptoms

All participants rated the presence of 15 upper-extremity symptoms (including hands) over the last week using a 5-point severity scale: 0 = none, 1 = mild, 2 = moderate, 3 = severe, and 4 = extreme.^34^ Symptoms included: swelling, pain, pain performing any specific activity, tingling, weakness, stiffness, poor range of movement, numbness, tightness, ache, heaviness, reddish skin coloring, tenderness, thickened/hardened skin, and hot areas on the skin. A 1-point change over time was considered clinically relevant.

#### Self-reported upper-extremity function

The 16-item Patient Reported Outcomes Measurement Information System (PROMIS) Bank v1.2 Upper-Extremity was used to measure upper-extremity function.^35^ This tool uses a 5-point rating scale to assess current ability to perform specific upper-body tasks, ranging from “unable to do” to “without any difficulty.”

#### Self-reported survivorship outcomes

The PROMIS-43 Profile v1.0 was used to assess fatigue, pain interference, physical function, sleep disturbance, anxiety, depression, and satisfaction with participation in social roles (6 items per domain, 5-point rating scale), plus pain intensity using a numeric rating scale (NRS 0-10 [no pain to worst imaginable pain]).^35^ All items used a 7-day recall.

For all PROMIS tools (apart from pain intensity), raw scores were converted to a *T*-score metric, in which 50 is the mean of a general population with an SD of 10. Higher scores reflect more of the domain being measured, which could be either negative (eg, more fatigue) or positive (eg, better physical function). For pain intensity, values were not converted, with change ≥2 considered a minimal important change (MIC)^36^ (the smallest change that patients consider important).^37^^,^^38^ The minimal important change for all other PROMIS outcomes was a change score ≥3.^37^^,^^38^

### Statistical analysis

Analyses were conducted in R.^39^ Continuous variables are reported as mean (95% CI) for normally distributed data, and median (range) for nonparametric data; categorical variables are presented as counts (percentages). For each lymphedema-related symptom, the proportion of participants reporting none, mild, moderate, severe, or extreme severity was calculated at baseline and 12 weeks. Between these timepoints, participants were classified as “improvers” (decrease ≥1 point), “decliners” (increase ≥1 point), or “maintainers” (no change). The number of symptoms reported (none, 1, 2, ≥3) was also calculated for each exercise group at both timepoints. Finally, a composite score of all lymphedema-related arm symptoms per participant was computed and reported as median (95% CI).

Group differences in symptom change were tested with χ^2^, and time, group, and time×group effects for PROMIS outcomes were evaluated using generalized estimating equations under an intention-to-treat, 2-sided framework (*P* < .05 deemed statistically significant). Based on previously described benchmarks,^35^^,^^36^^,^^38^^,^^40^^,^^41^ the proportion of participants reporting normal, mild, moderate or severe severity was calculated for each outcome at baseline and 12 weeks. To explore the effect of LOW vs HIGH supervision levels in persons with lymphedema, descriptive analyses were performed in participants presenting with lymphedema at baseline (objective criterion or self-reported clinical diagnosis).

## Results

Sixty women were recruited, with 30 participants randomly allocated to each group. A detailed flow diagram describing recruitment and retention and personal, treatment and behavioral characteristics are reported elsewhere.^24^ Overall, groups were similar at baseline. Regarding lymphedema risk, about half of participants had ≥5 axillary lymph nodes removed (LOW 53% [*n* = 16] vs HIGH 50% [*n* = 15]). All in LOW vs 83% (*n* = 25) in HIGH had or were receiving chemotherapy, while 67% (*n* = 20) in LOW vs 83% (*n* = 25) in HIGH had or were receiving radiotherapy. Approximately two-thirds had a BMI ≥25 kg.m^−2^ (LOW 67% [*n* = 20] vs HIGH 70% [*n* = 21]). Both groups reported a median of 41 min of structured exercise per week. One participant in LOW dropped out within 2 weeks of study commencement (moved away) and 3 were lost to follow-up (time constraints [*n* = 1], disease progression [*n* = 1], and extreme treatment-related symptoms [*n* = 1]). One participant in HIGH did not provide 12-week data due to time constraints. No participants were lost to follow-up due to lymphedema-related issues.

### Outcomes of interest

Lymphedema point prevalence varied by assessment method (Table 1). At baseline, 20 participants had lymphedema based on objective and/or self-report of a clinical diagnosis. Of these, 14 women (24%; 8 LOW, 6 HIGH) reported a lymphedema diagnosis (median time since diagnosis = 13.4 months). All HIGH participants described recurrent lymphedema vs 1 in LOW (Table S1). According to objective assessment, prevalence was higher in HIGH at baseline vs LOW (not supported statistically). Overall, there was no evidence that change in lymphedema prevalence at follow-up differed according to group allocation.

**Table 1.** Point prevalence of breast cancer-related lymphedema (BCRL) at baseline and 12 weeks in participants of the LOW vs HIGH supervision exercise groups (*n* = 60).

	LOW		HIGH	
	Baseline, *n* = 30	12 weeks, *n* = 26	Baseline, *n* = 28	12 weeks, *n* = 29
Lymphedema definition	No. (%)	No. (%)	No. (%)	No. (%)
Self-reported “yes” to being diagnosed with lymphedema since breast cancer diagnosis^a^				
Any body region	8 (27)	9 (35)	6 (21)	8 (28)
Arm	8 (27)	8 (31)	6 (21)	8 (28)
Trunk	1 (3)	2 (8)^a^	2 (7)	2 (7)
Breast	0 (0)	1 (4)	2 (7)	2 (7)
Met threshold for BCRL based on unilateral or bilateral bioimpedance spectroscopy criteria^b^				
One arm	3 (10)	2 (8)^c^	7 (25)	5 (20)^d^
Both arms	1 (3)	1 (4)^c^	0 (0)	0 (0)^d^
BCRL based on meeting any of the above definitions				
Any definition	10 (33)	11 (42)	10 (33)	11 (38)

Abbreviations: HIGH = high supervision; LOW = low supervision.

a One of these cases was trunk only.

b For unilateral cases, BCRL defined as L-Dex >7.1; for bilateral cases (*n* = 4), BCRL defined as arm-leg ratio <1.044 and <1.088 on the dominant and nondominant side, respectively.

c Denominator = 24.

d Denominator = 25.

At baseline, most participants reported at least 3 mild or moderate+ lymphedema-associated symptoms (Table 2). By 12 weeks, both groups showed reductions in the proportion reporting ≥3 moderate+ symptoms and improvements in composite symptom scores. Between 12% and 42% of the LOW group and 21% and 52% of the HIGH group reported improvements across the 15 symptoms assessed. For 12 of 15 symptoms, the absolute difference in the proportion of improvers favored the HIGH group (1%-17%), though none were statistically significant (Tables 3 and S2). Three exceptions were observed: poor range of movement improved in 52% of HIGH vs 12% of LOW (*P* = .003), while heaviness and tenderness improvements slightly favored LOW (absolute differences 6% and 3%, respectively). Subgroup analyses of participants with baseline lymphedema showed similar patterns, though composite score improvements were attenuated (2.5 vs. ≥4 in the total population) (Tables 2 and S2-S4).

**Table 2.** Proportion of participants with at least mild and moderate-intensity, self-reported lymphedema-related arm symptoms in the LOW vs HIGH supervision exercise groups.

Number of symptoms	LOW		HIGH	
	Baseline, No. (%)	12 weeks, No. (%)	Baseline, No. (%)	12 weeks, No. (%)
≥Mild symptoms				
0	5 (17)	1 (4)	0 (0)	2 (7)
1	1 (3)	1 (4)	1 (3)	2 (7)
2	3 (10)	5 (19)	2 (7)	1 (3)
3+	21 (70)	19 (73)	27 (90)	24 (83)
≥Moderate symptoms				
0	12 (40)	12 (46)	7 (23)	13 (45)
1	1 (3)	3 (12)	4 (13)	3 (10)
2	1 (3)	1 (4)	0 (0)	3 (10)
3+	16 (53)	10 (39)	19 (63)	10 (34)
Composite score of all symptoms				
Median; 95% CI	25; 18 to 30	21; 19 to 30	27; 21 to 35	23; 20 to 30

Percentages do not always equal 100, due to rounding.

Abbreviations: HIGH = high supervision; LOW = low supervision.

**Table 3.** Proportion of self-reported lymphedema-related arm symptom severity at baseline and 12 weeks, and proportion of improvers, decliners, and maintainers over time in LOW vs HIGH supervision exercise groups.

Group	Severity of symptom	Baseline, No. (%)	12 weeks, No. (%)	Change from baseline, No. (%)		
				Improve	Maintain	Decline
Swelling						
LOW	None	21 (70)	16 (62)	6 (23)	16 (62)	4 (15)
	Mild	4 (13)	7 (27)			
	≥Moderate	5 (17)	3 (12)			
HIGH	None	14 (47)	17 (59)	7 (24)	20 (69)	2 (7)
	Mild	11 (37)	8 (28)			
	≥Moderate	5 (17)	4 (14)			
Pain						
LOW	None	12 (40)	10 (38)	5 (19)	17 (65)	4 (15)
	Mild	9 (30)	10 (38)			
	≥Moderate	9 (30)	6 (23)			
HIGH	None	10 (33)	11 (39)	10 (36)	12 (43)	6 (21)
	Mild	7 (23)	12 (43)			
	≥Moderate	13 (43)	5 (18)			
Pain from activity						
LOW	None	13 (48)	12 (50)	5 (21)	16 (67)	3 (13)
	Mild	3 (11)	3 (13)			
	≥Moderate	11 (41)	9 (38)			
HIGH	None	10 (36)	14 (54)	10 (38)	13 (50)	3 (12)
	Mild	4 (14)	6 (23)			
	≥Moderate	14 (50)	6 (23)			
Tingling						
LOW	None	13 (43)	14 (54)	8 (31)	15 (58)	3 (12)
	Mild	11 (37)	9 (35)			
	≥Moderate	6 (20)	3 (11)			
HIGH	None	11 (37)	14 (48)	11 (38)	15 (52)	3 (10)
	Mild	10 (33)	11 (38)			
	≥Moderate	9 (30)	4 (14)			
Weakness						
LOW	None	14 (47)	10 (38)	8 (31)	12 (46)	6 (23)
	Mild	7 (23)	7 (27)			
	≥Moderate	9 (30)	9 (35)			
HIGH	None	8 (27)	9 (31)	13 (45)	13 (45)	3 (10)
	Mild	10 (33)	16 (55)			
	≥Moderate	12 (40)	4 (14)			
Stiffness						
LOW	None	14 (47)	11 (42)	11 (42)	11 (42)	4 (15)
	Mild	6 (20)	11 (42)			
	≥Moderate	10 (33)	4 (16)			
HIGH	None	8 (27)	12 (41)	16 (55)	7 (24)	6 (21)
	Mild	9 (30)	11 (38)			
	≥Moderate	13 (43)	6 (21)			
Poor range of movement (*P* = .003)^a^						
LOW	None	14 (48)	7 (27)	3 (12)	13 (50)	10 (38)
	Mild	9 (31)	12 (46)			
	≥Moderate	6 (21)	7 (27)			
HIGH	None	9 (30)	17 (59)	15 (52)	11 (38)	3 (10)
	Mild	7 (23)	8 (28)			
	≥Moderate	14 (47)	4 (13)			
Numbness						
LOW	None	12 (41)	10 (40)	7 (28)	13 (52)	5 (20)
	Mild	8 (28)	9 (36)			
	≥Moderate	9 (31)	6 (24)			
HIGH	None	12 (40)	14 (48)	9 (31)	18 (62)	2 (7)
	Mild	8 (27)	9 (31)			
	≥Moderate	10 (33)	6 (21)			
Tightness						
LOW	None	11 (37)	12 (46)	9 (35)	12 (46)	5 (19)
	Mild	8 (27)	6 (23)			
	≥Moderate	11 (36)	8 (31)			
HIGH	None	6 (20)	14 (48)	13 (45)	14 (48)	2 (7)
	Mild	10 (33)	9 (31)			
	≥Moderate	14 (47)	6 (21)			
Ache						
LOW	None	14 (47)	15 (58)	9 (35)	13 (50)	4 (15)
	Mild	7 (23)	5 (19)			
	≥Moderate	9 (30)	6 (23)			
HIGH	None	10 (33)	10 (34)	11 (38)	11 (38)	7 (24)
	Mild	7 (23)	12 (41)			
	≥Moderate	13 (43)	7 (24)			
Heaviness						
LOW	None	16 (55)	16 (64)	10 (40)	14 (56)	1(4)
	Mild	6 (21)	6 (24)			
	≥Moderate	7 (24)	3 (12)			
HIGH	None	15 (50)	19 (66)	10 (34)	15 (52)	4 (14)
	Mild	6 (20)	7 (24)			
	≥Moderate	9 (30)	3 (10)			
Reddish skin coloring						
LOW	None	24 (80)	22 (85)	6 (23)	17 (65)	3 (12)
	Mild	4 (13)	2 (8)			
	≥Moderate	2 (7)	2 (8)			
HIGH	None	22 (74)	22 (76)	8 (28)	17 (59)	4 (14)
	Mild	4 (13)	7 (24)			
	≥Moderate	4 (13)	0 (0)			
Tenderness						
LOW	None	16 (53)	14 (54)	10 (39)	11 (42)	5 (19)
	Mild	8 (27)	8 (31)			
	≥Moderate	6 (20)	4 (15)			
HIGH	None	13 (45)	15 (54)	10 (36)	15 (54)	3 (11)
	Mild	5 (17)	11 (39)			
	≥Moderate	11 (38)	2 (7)			
Thickened hardened skin						
LOW	None	23 (77)	21 (81)	5 (19)	19 (73)	2 (8)
	Mild	3 (10)	5 (19)			
	≥Moderate	4 (13)	0 (0)			
HIGH	None	19 (66)	23 (82)	6 (21)	21 (75)	1 (4)
	Mild	5 (17)	4 (14)			
	≥Moderate	5 (17)	1 (4)			
Hot areas on the skin						
LOW	None	20 (67)	23 (88)	7 (27)	18 (69)	1 (4)
	Mild	6 (20)	3 (12)			
	≥Moderate	4 (13)	0 (0)			
HIGH	None	16 (55)	20 (71)	8 (29)	18 (64)	2 (7)
	Mild	8 (28)	7 (25)			
	≥Moderate	5 (17)	1 (4)			

Abbreviations: HIGH = high supervision; LOW = low supervision.

a Between group change (*P* < .05) favoring the HIGH group. Improvers (decreased ≥1 point), decliners (increased ≥1 point), maintainers (unchanged).

Clinically meaningful improvements were observed for fatigue, physical function, sleep disturbance, anxiety, depression, and satisfaction with social roles and activities for both groups (*P* < .05 for all except anxiety; Table 4). The HIGH group also showed improvements (*P* < .05) for upper-extremity function, pain interference, and pain intensity, although the latter did not reach the clinically relevant threshold. At baseline, at least one-third of participants in both groups reported moderate+ fatigue, pain interference, pain intensity and sleep disturbance (Table S5). For upper-extremity function, 50% of HIGH participants vs 33% of LOW participants reported moderate+ dysfunction, with 55% of HIGH and 24% of LOW showing improvement by 12 weeks (Table S5). Subgroup analyses of participants with baseline lymphedema showed similar patterns (Table S6).

**Table 4.** Mean severity of self-reported upper-extremity function and other breast cancer-relevant survivorship outcomes in the LOW vs HIGH supervision exercise groups (*n* = 60).

	Baseline			12 weeks			Change from baseline	
	No.	Mean	(95% CI)	No.	Mean	(95% CI)	Mean Δ	95% CI
Upper-extremity function (*P* = .017)								
LOW	30	45.3	(42.1 to 48.4)	25	46.3	(43.0 to 49.6)	1.0	(−0.5 to 2.5)
HIGH	30	41.0	(38.4 to 43.6)	29	45.5	(42.7 to 48.3)	4.5^a^^,^^b^^,^^c^	(2.0 to 7.0)
Fatigue								
Low	30	60.2	(57.0 to 63.4)	26	54.6	(51.7 to 57.5)	−5.7^b^^,^^d^	(−8.9 to −2.4)
HIGH	30	58.7	(55.7 to 61.7)	29	50.7	(47.7 to 53.7)	−7.9^b^^,^^d^	(−11.6 to −4.3)
Pain interference								
LOW	30	55.4	(52.0 to 58.8)	26	52.6	(49.5 to 55.8)	−2.8	(−5.8 to 0.1)
HIGH	30	57.1	(54.1 to 60.0)	29	51.4	(48.4 to 54.3)	−5.7^b^^,^^d^	(−8.8 to −2.7)
Pain intensity (NRS, 0-10) (*P* =.002)								
LOW	30	3.2	(2.5 to 4.0)	26	3.0	(2.2 to 3.8)	−0.2	(0.6 to −1.0)
HIGH	30	4.0	(3.1 to 4.9)	29	3.0	(2.2 to 3.7)	−1.1^a^	(−0.4 to −1.8)
Physical function								
LOW	30	43.1	(40.2 to 46.0)	26	47.4	(44.5 to 50.2)	4.2^b^^,^^d^	(2.8 to 5.6)
HIGH	30	40.4	(38.8 to 42.0)	29	46.3	(43.6 to 48.9)	5.9^b^^,^^d^	(3.7 to 8.1)
Sleep disturbance								
LOW	30	57.7	(54.6 to 60.7)	26	52.5	(49.1 to 55.9)	−5.2^b^^,^^d^	(−8.3 to −2.0)
HIGH	30	55.4	(53.5 to 57.4)	29	50.3	(47.8 to 52.8)	−5.1^b^^,^^d^	(−7.8 to −2.4)
Anxiety								
LOW	30	56.2	(52.9 to 59.6)	26	51.8	(48.0 to 55.6)	−4.4^b^	(−8.0 to 0.9)
HIGH	30	56.2	(53.5 to 58.8)	29	51.6	(48.7 to 54.6)	−4.5^b^^,^^d^	(−7.2 to −1.9)
Depression								
LOW	30	52.0	(48.6 to 55.3)	26	47.6	(44.7 to 50.5)	−4.4^b^^,^^d^	(−7.6 to −1.2)
HIGH	30	51.9	(48.7 to 66.1)	29	46.7	(44.0 to 49.5)	−5.2^b^^,^^d^	(−8.4 to −2.0)
Satisfaction with social roles and activities								
LOW	30	43.3	(39.8 to 46.9)	26	49.5	(46.7 to 52.3)	6.2^b^^,^^d^	(3.0 to 9.3)
HIGH	30	43.4	(40.7 to 46.1)	29	50.3	(47.2 to 53.3)	6.8^b^^,^^d^	(3.7 to 10.0)

Numeric rating scale using PROMIS Bank v1.2 Upper-Extremity and PROMIS-43 Profile v1.0. Higher scores reflect more of the domain being measured (eg, worse fatigue, better physical function).

Abbreviations: HIGH = high supervision; LOW = low supervision; PROMIS = Patient Reported Outcomes Measurement Information System; NRS = numeric rating scale.

a Between group change (*P* < .05) favoring the HIGH group.

b Minimal important change (MIC) from baseline (≥3).

c MIC between groups (≥3).^37^^,^^38^

d Within-group change (*P* < .05).

## Discussion

Overall, we found that participation in a 12-week, individually prescribed resistance and aerobic exercise program did not increase risk of, or exacerbate existing lymphedema, and was associated with improvements to lymphedema-related arm symptoms and other health outcomes relevant to breast cancer survivors, irrespective of supervision level. There was some evidence for superiority of benefit to range of movement, upper-extremity function, and pain interference and intensity through higher levels of supervision. However, more work is required before these findings can be considered definitive, as they come from analyses of secondary outcomes and need confirmation in adequately powered trials.

Breast cancer-related lymphedema results from a meta-analysis (2022) showed trends favoring any/all levels of exercise supervision in the prevention and treatment of lymphedema.^18^ In line with these results, point prevalence data from the present study suggest that level of supervision did not impact BCRL development. Further, both groups experienced improvements or no worsening in most arm symptoms. These findings are consistent with previously reported results from the SAFE trial, which found both supervision levels safe (no serious adverse events) and feasible (both groups exceeded exercise target).^24^ Superior improvements in range of movement were seen in the HIGH group. However, baseline data suggest that range of movement was worse in the HIGH group, with a greater potential for improvement when compared with the LOW group. While higher supervision may better address specific lymphedema-related issues like poor range of movement by ensuring proper technique and intensity monitoring, overall results support exercise delivery at both supervision levels for managing lymphedema risk and associated symptoms.

Beyond lymphedema and its symptoms, both groups showed meaningful improvements across most outcomes. Superior improvements in upper-extremity function and pain interference were observed in the HIGH group; however, baseline differences between groups likely influenced capacity for improvement in the LOW group. As such, indication of superiority of the HIGH group should be interpreted with caution. In the primary SAFE trial publication,^24^ higher supervision improved self-efficacy and muscular strength, with improvements in both groups in quality of life, fitness, and cost-effectiveness.^25^ Combined with the current findings focused on treatment-related side effects, the overall results support the value of exercise—even at lower supervision levels, while suggesting potential for additional or superior benefits with higher levels of supervision.

One-third (*n* = 20) of participants had BCRL at baseline, with most reporting moderate symptoms that improved or remained stable, regardless of supervision allocation. We also found that more than 2 in 3 of these women reported improvement in fatigue and pain interference. These findings align with prior studies involving women with BCRL, all of which consistently support that exercise does not worsen BCRL and with potential for improving other relevant survivorship issues.^17^^,^^18^ Nonetheless, larger trials are warranted to confirm these findings and whether the effect of supervision level is modified by the stage of lymphedema. Also, while most participants maintained or improved arm symptoms and survivorship outcomes, a minority of participants in both groups experienced worsening of outcomes, highlighting the importance of individual symptom response monitoring with appropriate exercise modification.

This study has several limitations. First, this paper reports on analyses of secondary outcomes. The sample size is small (especially for the BCRL subgroup) and groups showed imbalance for some outcomes at baseline, potentially swaying results in favor of the HIGH group. Also, this trial did not involve a control group, introducing the possibility that observed changes could be due to the passage of time or engagement with an allied health professional rather than the interventions. Nevertheless, the sample comprised insufficiently active women, with high compliance to the intervention (physical activity increased by 244 min.week^−1^ from baseline),^24^ suggesting improvements are likely reflective of intervention effects. Finally, while the method of BCRL measurement influences point prevalence rates (in this study and others previously),^8^^,^^42-44^ we observed similar trends at follow-up in outcomes of interest between the HIGH and LOW groups for any given measurement method.

Strengths of the study include the randomized design, intention-to-treat analyses, blinded assessment and the use of well-validated questionnaires, and incorporation of both objective and subjective assessments of BCRL. Further, at baseline, at least one-third of the sample reported moderate+ severity for most health outcomes and over half presented with 3 or more moderate+ arms symptoms. This attests to the ability of the eligibility criteria to identify breast cancer survivors with persistent and complex health concerns, often excluded or absent from exercise trials. About half of the participants had ≥5 axillary lymph nodes removed and at least two-thirds had a BMI ≥25 kg.m^−2^, all were insufficiently active, and all had received or were receiving, chemotherapy and/or radiotherapy. Considering known risk factors for developing BCRL, the results of this study are likely generalizable to those at higher risk for developing this condition. Finally, the present study was novel, providing a much-needed head-to-to head comparison of exercise supervision levels, of importance for clinical practice and policy makers.

Overall, while future studies with larger sample sizes are warranted to confirm these findings, results support that breast cancer survivors at risk of, or living with, lymphedema can benefit through participation in exercise, including when supervision is limited. Further, results provide confidence that national and international exercise guidelines promoted to the wider population are likely generalizable to breast cancer survivors previously underrepresented in exercise oncology studies. Finally, results of this study are particularly encouraging for individuals facing barriers to supervised exercise as they support more equitable and patient-centered delivery models that may benefit the growing number of breast cancer survivors and potentially broaden population impact.

## Supplementary Material

pkag004_Supplementary_Data

## Data Availability

The data underlying this article are available in the article and in the Supplementary Material. De-identified data, dependent variables, and participant characteristics may be provided upon reasonable request to the author.
