# Supplementary material for: Comparison of the effects of low- versus high-supervision exercise on breast cancer survivorship outcomes
Source: JNCI Cancer Spectr. 2026 Jan 25;10(1):pkag004. doi: 10.1093/jncics/pkag004 (PMC12928991; doi:10.1093/jncics/pkag004)
Supplement: pkag004_Supplementary_Data [file pkag004_supplementary_data.zip › SAFE supp files 1 Dec.docx]

**Supplementary File**

**Supplementary Methods:** Bioimpedance spectroscopy positioning protocol

Participants were positioned in supine with arms and legs slightly abducted from the trunk with palms facing down. Two measurement electrodes were placed on the wrist (for arm measures) and ankle (for leg measures) with a current drive electrode placed approximately 10 cm proximal to the base of the middle finger or toe, respectively, and another placed on the ipsilateral or contralateral foot or hand, respectively. In participants with unilateral axillary surgery, the ratio of impedance comparing the treated and untreated sides was calculated and converted into a lymphedema index (L-Dex) score. L-Dex >7.1 was defined as BCRL ^32^. For participants with bilateral surgery, arm to leg impedance ratios were calculated. Impedance ratios <1.044 (dominant-side) and <1.080 (non-dominant-side) were considered indicative of BCRL ^33^.

**Table S1**. Lymphedema descriptives for participants who answered “yes” to having a clinical diagnosis of lymphedema at baseline.

|  | ALL  n = 14  n (%) | LOW  n = 8  n (%) | HIGH  n = 6  n (%) |
| --- | --- | --- | --- |
| Type of lymphedema ^a^ | | | |
| Single episode | 2 (15) | 2 (29) | 0 (0) |
| Recurrent | 7 (54) | 1 (14) | 6 (100) |
| Persistent | 4 (31) | 4 (57) | 0 (0) |
| Severity of lymphedema | | | |
| Mild | 7 (50) | 4 (50) | 3 (50) |
| Moderate | 1 (7) | 1 (13) | 0 (0) |
| Severe | 0 (0) | 0 (0) | 0 (0) |
| Unsure | 6 (43) | 3 (38) | 3 (50) |
| Pitting | | | |
| Non-pitting | 6 (43) | 4 (50) | 2 (33) |
| Pitting | 0 (0) | 0 (0) | 0 (0) |
| Unsure | 8 (57) | 4 (50) | 4 (67) |
| Both | 0 (0) | 0 (0) | 0 (0) |
| Lymphedema stage | | | |
| Stage 0 | 2 (14) | 1 (13) | 1 (17) |
| Stage 1 | 2 (14) | 2 (25) | 0 (0) |
| Stage 2 | 0 (0) | 0 (0) | 0 (0) |
| Stage 3 | 0 (0) | 0 (0) | 0 (0) |
| Unsure | 10 (71) | 5 (63) | 5 (83) |
| Currently have lymphedema | | | |
| Yes | 8 (57) | 6 (75) | 2 (33) |
| No | 6 (43) | 2 (25) | 4 (67) |
| Time since lymphedema diagnosis (Median, 95% CI)^b^ | | | |
| Days | 409 (221 to 997) | 409 (157 to 997) | 400 (221 to 6240) |

Percentages do not always equal 100, due to rounding. ^a^ Due to missing data (ALL n = 13, LOW n = 7); ^b^ Due to missing data (LOW n = 7, HIGH n = 4).

**Table S2**. Median severity of lymphedema-related arm symptoms at baseline, 12 weeks and change over time in the LOW versus HIGH supervision exercise groups.

| *TOTAL POPULATION* | | | | | | |
| --- | --- | --- | --- | --- | --- | --- |
|  | Baseline | | 12 weeks | | Change from baseline | |
|  | n | Median (95% CI) | n | Median (95% CI) | n | Median (95% CI) |
| Swelling | | | | | | |
| LOW | 30 | 1 (1 to 1) | 26 | 1 (1 to 2) | 26 | 0 (0 to 0) |
| HIGH | 30 | 2 (1 to 2) | 29 | 1 (1 to 2) | 29 | 0 (0 to 0) |
| Pain | | | | | | |
| LOW | 30 | 2 (1 to 2) | 26 | 2 (1 to 2) | 26 | 0 (0 to 0) |
| HIGH | 30 | 2 (1 to 3) | 28 | 2 (1 to 2) | 28 | 0 (-1 to 0) |
| Pain during activity | | | | | | |
| LOW | 27 | 2 (1 to 3) | 26 | 1.5 (1 to 3) | 24 | 0 (0 to 0) |
| HIGH | 28 | 2.5 (1 to 3) | 28 | 1 (1 to 2) | 26 | 0 (-1 to 0) |
| Tingling | | | | | | |
| LOW | 30 | 2 (1 to 2) | 26 | 1 (1 to 2) | 26 | 0 (-1 to 0) |
| HIGH | 30 | 2 (1 to 2) | 29 | 2 (1 to 2) | 29 | 0 (-1 to 0) |
| Weakness | | | | | | |
| LOW | 30 | 2 (1 to 2) | 26 | 2 (1 to 3) | 26 | 0 (-1 to 0) |
| HIGH | 30 | 2 (2 to 3) | 29 | 2 (1 to 2) | 29 | 0 (-1 to 0) |
| Stiffness | | | | | | |
| LOW | 30 | 2 (1 to 3) | 26 | 2 (1 to 2) | 26 | 0 (-1 to 0) |
| HIGH | 30 | 2 (2 to 3) | 29 | 2 (1 to 2) | 29 | -1 (-1 to 0)^#^ |
| Poor range of movement | | | | | | |
| LOW | 29 | 2 (1 to 2) | 26 | 2 (2 to 2) | 26 | 0 (0 to 1) |
| HIGH | 30 | 2 (2 to 3) | 29 | 1 (1 to 2) | 29 | -1 (-1 to 0)^#^ |
| Numbness | | | | | | |
| LOW | 29 | 2 (1 to 3) | 26 | 2 (1 to 2) | 25 | 0 (0 to 0) |
| HIGH | 30 | 2 (1 to 3) | 29 | 2 (1 to 2) | 29 | 0 (-1 to 0) |
| Tightness | | | | | | |
| LOW | 30 | 2 (1 to 3) | 26 | 2 (1 to 3) | 26 | 0 (-1 to 0) |
| HIGH | 30 | 2 (2 to 3) | 29 | 2 (1 to 2) | 29 | 0 (-1 to 0) |
| Ache | | | | | | |
| LOW | 30 | 2 (1 to 2) | 26 | 1 (1 to 2) | 26 | 0 (-1 to 0) |
| HIGH | 30 | 2 (1 to 3) | 29 | 2 (1 to 2) | 29 | 0 (-1 to 0) |
| Heaviness | | | | | | |
| LOW | 29 | 1 (1 to 2) | 26 | 1 (1 to 2) | 25 | 0 (-1 to 0) |
| HIGH | 30 | 1.5 (1 to 2) | 29 | 1 (1 to 2) | 29 | 0 (-1 to 0) |
| Reddish skin color | | | | | | |
| LOW | 30 | 1 (1 to 1) | 26 | 1 (1 to 1) | 26 | 0 (0 to 0) |
| HIGH | 30 | 1 (1 to 1) | 29 | 1 (1 to 1) | 29 | 0 (0 to 0) |
| Tenderness | | | | | | |
| LOW | 30 | 1 (1 to 2) | 26 | 1 (1 to 2) | 26 | 0 (-1 to 0) |
| HIGH | 29 | 2 (1 to 3) | 29 | 1 (1 to 2) | 28 | 0 (-1 to 0) |
| Thickened skin | | | | | | |
| LOW | 30 | 1 (1 to 1) | 26 | 1 (1 to 1) | 26 | 0 (0 to 0) |
| HIGH | 29 | 1 (1 to 2) | 29 | 1 (1 to 1) | 28 | 0 (0 to 0) |
| Hot areas on skin | | | | | | |
| LOW | 30 | 1 (1 to 2) | 26 | 1 (1 to 1) | 26 | 0 (0 to 0) |
| HIGH | 29 | 1 (1 to 2) | 29 | 1 (1 to 1) | 28 | 0 (0 to 0) |
| *SUBPOPULATION OF PARTICIPANTS WITH LYMPHEDEMA AT BASELINE^a^* | | | | | | |
| Swelling | | | | | | |
| LOW | 10 | 2 (1 to 3) | 9 | 2 (1 to 3) | 9 | -1 (-1 to 1)^#^ |
| HIGH | 10 | 2 (1 to 3) | 10 | 2 (1 to 3) | 10 | 0 (-1 to 0) |
| Pain (NRS) | | | | | | |
| LOW | 10 | 1.5 (1 to 3) | 9 | 2 (1 to 3) | 9 | 0 (-1 to 1) |
| HIGH | 10 | 2.5 (1 to 3) | 9 | 1 (1 to 3) | 9 | 0 (-1 to 0) |
| Pain during activity | | | | | | |
| LOW | 9 | 2 (1 to 4) | 9 | 2 (1 to 4) | 9 | 0 (-1 to 0) |
| HIGH | 9 | 2 (1 to 3) | 10 | 1 (1 to 3) | 9 | 0 (-1 to 0) |
| Tingling | | | | | | |
| LOW | 10 | 2 (1 to 3) | 9 | 2 (1 to 4) | 9 | 0 (-1 to 1) |
| HIGH | 10 | 2 (1 to 3) | 10 | 1.5 (1 to 3) | 10 | 0 (-1 to 0) |
| Weakness | | | | | | |
| LOW | 10 | 2.5 (1 to 3) | 9 | 2 (1 to 3) | 9 | 0 (-1 to 1) |
| HIGH | 10 | 2 (1 to 3) | 10 | 2 (1, 3) | 10 | 0 (-1 to 1) |
| Stiffness | | | | | | |
| LOW | 10 | 1.5 (1 to 4) | 9 | 2 (1 to 2) | 9 | 0 (-1 to 0) |
| HIGH | 10 | 2.5 (1 to 3) | 10 | 2 (1 to 3) | 10 | -0.5 (-1 to 1) |
| Poor range of movement | | | | | | |
| LOW | 10 | 2 (1 to 3) | 9 | 2 (1 to 3) | 9 | 0 (-1 to 1) |
| HIGH | 10 | 2.5 (2 to 3) | 10 | 2 (1 to 3) | 10 | -1 (-1 to 1)^#^ |
| Numbness | | | | | | |
| LOW | 10 | 2.5 (1 to 3) | 9 | 2 (1 to 3) | 9 | 0 (-1 to 0) |
| HIGH | 10 | 2.5 (1 to 3) | 10 | 2 (1 to 3) | 10 | -0.5 (-1 to 0) |
| Tightness | | | | | | |
| LOW | 10 | 2 (1 to 3) | 9 | 2 (1 to 4) | 9 | 0 (-1 to 1) |
| HIGH | 10 | 3 (2 to 3) | 10 | 2 (1 to 3) | 10 | -1 (-2 to 0)^#^ |
| Ache | | | | | | |
| LOW | 10 | 1.5 (1 to 4) | 9 | 1 (1 to 3) | 9 | 0 (-1 to 0) |
| HIGH | 10 | 2.5 (1 to 4) | 10 | 2 (1 to 3) | 10 | 0 (-1 to 0) |
| Heaviness | | | | | | |
| LOW | 10 | 2 (1 to 4) | 9 | 1 (1 to 3) | 9 | -1 (-1 to 0)^#^ |
| HIGH | 10 | 2 (1 to 3) | 10 | 1.5 (1 to 3) | 10 | 0 (-2 to 0) |
| Reddish skin color | | | | | | |
| LOW | 10 | 1 (1 to 2) | 9 | 1 (1 to 3) | 9 | 0 (-1 to 2) |
| HIGH | 10 | 1 (1 to 2) | 10 | 1 (1 to 1) | 10 | 0 (-1 to 0) |
| Tenderness | | | | | | |
| LOW | 10 | 1.5 (1 to 4) | 9 | 1 (1 to 3) | 9 | 0 (-1 to 0) |
| HIGH | 10 | 1 (1 to 3) | 10 | 1 (1 to 3) | 10 | 0 (-1 to 0) |
| Thickened skin | | | | | | |
| LOW | 10 | 1 (1 to 3) | 9 | 1 (1 to 2) | 9 | 0 (-1 to 0) |
| HIGH | 10 | 1 (1 to 3) | 10 | 1 (1 to 2) | 10 | 0 (-2 to 0) |
| Hot areas on skin | | | | | | |
| LOW | 10 | 1 (1 to 2) | 9 | 1 (1 to 2) | 9 | 0 (-1 to 0) |
| HIGH | 10 | 1 (1 to 3) | 10 | 1 (1 to 2) | 10 | 0 (0 to 0) |

^a^Lymphedema (L-Dex > 7.1 or bilateral criterion (as described in text) or self-reported clinical diagnosis).

^#^ indicates reduction in symptom severity from baseline considered clinically relevant.

**Table S3**. Proportion of participants with at least mild and moderate, self-reported lymphedema-related arm symptoms in the LOW versus HIGH supervision exercise groups in participants with lymphedema at baseline^a^

| Number of  symptoms | LOW | | | HIGH | | | |
| --- | --- | --- | --- | --- | --- | --- | --- |
|  | Baseline  n (%) | | 12 weeks  n (%) | Baseline  n (%) | | | 12 weeks  n (%) |
| ≥ Mild severity | | | | | | | |
| 0 | 0 (0) | | 1 (10) | 0 (0) | | | 2 (20) |
| 1 | 1 (10) | | 0 (0) | 1 (10) | | | 0 (0) |
| 2 | 2 (20) | | 2 (20) | 0 (0) | | | 0 (0) |
| 3+ | 7 (70) | | 7 (70) | 9 (90) | | | 8 (80) |
| ≥ Moderate severity | | | | | | | |
| 0 | 3 (30) | 3 (30) | | | 1 (10) | 3 (30) | |
| 1 | 1 (10) | 2 (20) | | | 1 (10) | 2 (20) | |
| 2 | 1 (10) | 1 (10) | | | 0 (0) | 1 (10) | |
| 3+ | 5 (50) | 4 (40) | | | 8 (80) | 4 (40) | |
| Composite score of all symptoms | | | | | | | |
| Median; 95% CI | 25.5; 17 to 46 | 23.0; 17 to 37 | | | 30.0; 19 to 40 | 27.5; 15 to 37 | |

^a^Lymphedema (L-Dex > 7.1 or bilateral criterion (as described in text) or self-reported clinical diagnosis).

**Table S4**. Proportion of self-reported lymphedema-related arm symptom severity at baseline, and proportion of improvers, decliners and maintainers over time in the LOW versus HIGH supervision exercise groups, in participants with lymphedema at baseline^a^

| Group | Severity  of arm symptoms | Baseline  n (%) | 12 weeks  n (%) | Change in symptom severity  from baseline^#^, n (%) | | | |  |
| --- | --- | --- | --- | --- | --- | --- | --- | --- |
|  |  |  |  | Improve | Maintain | | Decline |  |
| Swelling | | |  |  | | | |  |
| LOW | None | 3 (30) | 3 (33) | 5 (56) | 2 (22) | 2 (22) | |  |
|  | Mild | 3 (30) | 3 (33) |  |  |  |  |  |
|  | ≥Moderate | 4 (40) | 3 (33) |  |  |  |  |  |
| HIGH | None | 3 (30) | 4 (40) | 2 (20) | 7 (70) | 1 (10) | |  |
|  | Mild | 5 (50) | 4 (40) |  |  |  |  |  |
|  | ≥Moderate | 2 (20) | 2 (20) |  |  |  |  |  |
| Pain | | |  |  | | | | |
| LOW | None | 5 (50) | 4 (44) | 2 (22) | 5 (56) | 2 (22) | |  |
|  | Mild | 2 (20) | 3 (33) |  |  |  |  |  |
|  | ≥Moderate | 3 (30) | 2 (22) |  |  |  |  |  |
| HIGH | None | 4 (40) | 5 (56) | 3 (33) | 6 (67) | 0 (0) | |  |
|  | Mild | 1 (10) | 2 (22) |  |  |  |  |  |
|  | ≥Moderate | 4 (40) | 2 (22) |  |  |  |  |  |
| Pain from activity | | |  |  | | | | |
| LOW | None | 4 (44) | 4 (44) | 2 (22) | 6 (67) | 1 (11) | |  |
|  | Mild | 1 (11) | 1 (11) |  |  |  |  |  |
|  | ≥Moderate | 4 (44) | 4 (44) |  |  |  |  |  |
| HIGH | None | 4 (44) | 5 (56) | 2 (22) | 6 (67) | 1 (11) | |  |
|  | Mild | 1 (11) | 1 (11) |  |  |  |  |  |
|  | ≥Moderate | 4 (44) | 3 (33) |  |  |  |  |  |
| Tingling | | |  |  | | | | |
| LOW | None | 2 (20) | 3 (33) | 4 (44) | 3 (33) | 2 (22) | |  |
|  | Mild | 4 (40) | 4 (44) |  |  |  |  |  |
|  | ≥Moderate | 4 (40) | 2 (22) |  |  |  |  |  |
| HIGH | None | 3 (30) | 5 (50) | 4 (40) | 6 (60) | 0 (0) | |  |
|  | Mild | 3 (30) | 3 (30) |  |  |  |  |  |
|  | ≥Moderate | 4 (40) | 2 (20) |  |  |  |  |  |
| Weakness | | |  |  | | | | |
| LOW | None | 4 (40) | 3 (33) | 3 (33) | 4 (44) | 2 (22) | |  |
|  | Mild | 1 (10) | 2 (22) |  |  |  |  |  |
|  | ≥Moderate | 5 (50) | 4 (44) |  |  |  |  |  |
| HIGH | None | 3 (30) | 2 (20) | 4 (40) | 4 (40) | 2 (20) | |  |
|  | Mild | 3 (30) | 6 (60) |  |  |  |  |  |
|  | ≥Moderate | 4 (40) | 2 (20) |  |  |  |  |  |
| Stiffness | | |  |  | | | | |
| LOW | None | 5 (50) | 4 (44) | 4 (44) | 4 (44) | 1 (11) | |  |
|  | Mild | 1 (10) | 4 (44) |  |  |  |  |  |
|  | ≥Moderate | 4 (40) | 1 (11) |  |  |  |  |  |
| HIGH | None | 2 (20) | 3 (30) | 5 (50) | 3 (30) | 2 (20) | |  |
|  | Mild | 3 (30) | 5 (50) |  |  |  |  |  |
|  | ≥Moderate | 5 (50) | 2 (20) |  |  |  |  |  |
| Poor range of movement | | |  |  | | | | |
| LOW | None | 4 (40) | 3 (33) | 2 (22) | 4 (44) | 3 (33) | |  |
|  | Mild | 4 (40) | 4 (44) |  |  |  |  |  |
|  | ≥Moderate | 2 (20) | 2 (22) |  |  |  |  |  |
| HIGH | None | 1 (10) | 4 (40) | 6 (60) | 2 (20) | 2 (20) | |  |
|  | Mild | 4 (40) | 3 (30) |  |  |  |  |  |
|  | ≥Moderate | 5 (50) | 3 (30) |  |  |  |  |  |
| Numbness | | |  |  | | | | |
| LOW | None | 2 (20) | 2 (22) | 3 (33) | 5 (56) | 1 (11) | |  |
|  | Mild | 3 (30) | 4 (44) |  |  |  |  |  |
|  | ≥Moderate | 5 (50) | 3 (33) |  |  |  |  |  |
| HIGH | None | 4 (40) | 4 (40) | 5 (50) | 4 (40) | 1 (10) | |  |
|  | Mild | 1 (10) | 4 (40) |  |  |  |  |  |
|  | ≥Moderate | 5 (50) | 2 (20) |  |  |  |  |  |
| Tightness | | |  |  | | | | |
| LOW | None | 5 (50) | 3 (33) | 2 (22) | 4 (44) | 3 (33) | |  |
|  | Mild | 0 (0) | 3 (33) |  |  |  |  |  |
|  | ≥Moderate | 5 (50) | 3 (33) |  |  |  |  |  |
| HIGH | None | 1 (10) | 4 (40) | 6 (60) | 3 (30) | 1 (10) | |  |
|  | Mild | 2 (20) | 3 (30) |  |  |  |  |  |
|  | ≥Moderate | 7 (70) | 3 (30) |  |  |  |  |  |
| Ache | | | | | | | | |
| LOW | None | 5 (50) | 5 (55) | 2 (22) | 6 (67) | 1 (11) | |  |
|  | Mild | 3 (30) | 2 (22) |  |  |  |  |  |
|  | ≥Moderate | 2 (20) | 2 (22) |  |  |  |  |  |
| HIGH | None | 3 (30) | 4 (40) | 4 (40) | 6 (60) | 0 (0) | |  |
|  | Mild | 2 (20) | 3 (30) |  |  |  |  |  |
|  | ≥Moderate | 5 (50) | 3 (30) |  |  |  |  |  |
| Heaviness | | |  |  | | | | |
| LOW | None | 4 (40) | 5 (56) | 5 (56) | 4 (44) | 0 (0) | |  |
|  | Mild | 3 (30) | 2 (22) |  |  |  |  |  |
|  | ≥Moderate | 3 (30) | 2 (22) |  |  |  |  |  |
| HIGH | None | 4 (40) | 5 (50) | 4 (40) | 5 (50) | 1 (10) | |  |
|  | Mild | 2 (20) | 3 (30) |  |  |  |  |  |
|  | ≥Moderate | 4 (40) | 2 (20) |  |  |  |  |  |
| Reddish skin coloring | | |  |  | | | | |
| LOW | None | 8 (80) | 5 (56) | 2 (22) | 4 (44) | 3 (33) | |  |
|  | Mild | 1 (10) | 2 (22) |  |  |  |  |  |
|  | ≥Moderate | 1 (10) | 2 (22) |  |  |  |  |  |
| HIGH | None | 7 (70) | 9 (90) | 3 (30) | 7 (70) | 0 (0) | |  |
|  | Mild | 2 (20) | 1 (10) |  |  |  |  |  |
|  | ≥Moderate | 1 (10) | 0 (0) |  |  |  |  |  |
| Tenderness | | |  |  | | | | |
| LOW | None | 5 (50) | 5 (56) | 3 (33) | 6 (67) | 0 (0) | |  |
|  | Mild | 2 (20) | 2 (22) |  |  |  |  |  |
|  | ≥Moderate | 3 (30) | 2 (22) |  |  |  |  |  |
| HIGH | None | 6 (60) | 6 (60) | 2 (20) | 7 (70) | 1 (10) | |  |
|  | Mild | 1 (10) | 2 (20) |  |  |  |  |  |
|  | ≥Moderate | 3 (30) | 2 (20) |  |  |  |  |  |
| Thickened hardened skin | | |  |  | | | | |
| LOW | None | 7 (70) | 6 (67) | 2 (22) | 6 (67) | 1 (11) | |  |
|  | Mild | 1 (10) | 3 (33) |  |  |  |  |  |
|  | ≥Moderate | 2 (20) | 0 (0) |  |  |  |  |  |
| HIGH | None | 7 (70) | 8 (80) | 2 (20) | 7 (70) | 1 (10) | |  |
|  | Mild | 0 (0) | 1 (10) |  |  |  |  |  |
|  | ≥Moderate | 3 (30) | 1 (10) |  |  |  |  |  |
| Hot areas on the skin | | |  |  | | | | |
| LOW | None | 6 (60) | 7 (78) | 2 (22) | 6 (67) | 1 (11) | |  |
|  | Mild | 3 (30) | 2 (22) |  |  |  |  |  |
|  | ≥Moderate | 1 (10) | 0 (0) |  |  |  |  |  |
| HIGH | None | 7 (70) | 6 (60) | 1 (20) | 8 (80) | 1 (10) | |  |
|  | Mild | 1 (10) | 3 (30) |  |  |  |  |  |
|  | ≥Moderate | 2 (20) | 1 (10) |  |  |  |  |  |

^a^ Lymphedema (L-Dex > 7.1 or bilateral criterion (as described in text) or self-reported clinical diagnosis). Severe and extreme severity have been combined with moderate as case numbers were low/none. ^#^ Improvers (decreased ≥ 1 point), Decliners (increased ≥ 1 point), and Maintainers (unchanged) based on change scores between baseline and 12-weeks.

**Table S5**. Severity of self-reported upper-extremity function and breast cancer-relevant survivorship outcomes at baseline and 12 weeks, and proportion of improvers, decliners and maintainers over time in the LOW versus HIGH supervision exercise groups.

| Group | PROMIS outcomes with severity benchmarks | Baseline | 12 weeks^a^ | Change in severity  from baseline (n (%)) | | |
| --- | --- | --- | --- | --- | --- | --- |
|  |  | n (%) | n (%) | Improve | Maintain | Decline |
| Upper-extremity function ^e^ (n =1 missing LOW) | | | | | | |
| LOW | Normal (> 45) | 13 (43) | 11 (44) | 6 (24) | 18 (72) | 1 (4) |
|  | Mild dysfunction (40-45) | 7 (23) | 6 (24) |  |  |  |
|  | Moderate dysfunction (39-30) | 9 (30) | 7 (28) |  |  |  |
|  | Severe dysfunction (<30) | 1 (3) | 1 (4) |  |  |  |
| HIGH | Normal (> 45) | 7 (23) | 12 (41) | 16 (55) | 12 (41) | 1 (3) |
|  | Mild dysfunction (40-45) | 8 (27) | 9 (31) |  |  |  |
|  | Moderate dysfunction (39-30) | 14 (47) | 8 (28) |  |  |  |
|  | Severe dysfunction (<30) | 1 (3) | 0 (0) |  |  |  |
| Fatigue ^b^ | | | | | | |
| LOW | Normal (<50) | 4 (13) | 6 (23) | 17 (65) | 5 (19) | 4 (15) |
|  | Mild (50–54) | 1 (3) | 5 (19) |  |  |  |
|  | Moderate (55–74) | 25 (83) | 15 (58) |  |  |  |
| HIGH | Normal (<50) | 4 (13) | 13 (45) | 20 (69) | 6 (21) | 3 (10) |
|  | Mild (50–54) | 5 (17) | 7 (24) |  |  |  |
|  | Moderate (55–74) | 20 (67) | 9 (31) |  |  |  |
|  | Severe (≥75) | 1 (3) | 0 (0) |  |  |  |
| Pain interference ^b^ | | | | | | |
| LOW | Normal (<50) | 7 (23) | 9 (35) | 15 (58) | 6 (23) | 5 (19) |
|  | Mild (50–59) | 14 (47) | 11 (42) |  |  |  |
|  | Moderate (60–69) | 7 (23) | 6 (23) |  |  |  |
|  | Severe (≥70) | 2 (7) | 0 (0) |  |  |  |
| HIGH | Normal (<50) | 5 (17) | 10 (34) | 20 (69) | 7 (24) | 2 (7) |
|  | Mild (50–59) | 12 (40) | 15 (52) |  |  |  |
|  | Moderate (60–69) | 12 (40) | 4 (14) |  |  |  |
|  | Severe (≥70) | 1 (3) | 0 (0) |  |  |  |
| Pain NRS (0-10) ^c^ | | | | | | |
| LOW | None 0 | 4 (13) | 3 (12) | 4 (15) | 16 (62) | 6 (23) |
|  | Mild (1-3) | 14 (47) | 12 (46) |  |  |  |
|  | Moderate (4-6) | 10 (33) | 10 (38) |  |  |  |
|  | Severe ≥7 | 2 (7) | 1 (4) |  |  |  |
| HIGH | None 0 | 1 (3) | 3 (10) | 12 (41) | 14 (48) | 3 (10) |
|  | Mild (1-3) | 14 (47) | 17 (59) |  |  |  |
|  | Moderate (4-6) | 8 (27) | 6 (21) |  |  |  |
|  | Severe ≥7 | 7 (23) | 3 (10) |  |  |  |
| Physical function ^d^ | | | | | | |
| LOW | Normal (>50) | 5 (17) | 8 (31) | 14 (54) | 12 (46) | 0 (0) |
|  | Mild dysfunction (35-50) | 20 (67) | 18 (69) |  |  |  |
|  | Mod. dysfunction (<35 - 20) | 5 (17) | 0 (0) |  |  |  |
| HIGH | Normal (>50) | 0 (0) | 6 (21) | 19 (66) | 7 (24) | 3 (10) |
|  | Mild dysfunction (35-50) | 25 (83) | 21 (72) |  |  |  |
|  | Mod. dysfunction (<35 - 20) | 5 (17) | 2 (7) |  |  |  |
| Sleep disturbance^d^ | | | | | | |
| LOW | Normal (<45) | 2 (7) | 7 (27) | 13 (50) | 10 (38) | 3 (11) |
|  | Mild (45-55) | 10 (33) | 9 (35) |  |  |  |
|  | Moderate (>55-60) | 3 (10) | 4 (15) |  |  |  |
|  | Severe (>60) | 15 (50) | 6 (23) |  |  |  |
| HIGH | Normal (<45) | 1 (3) | 8 (28) | 20 (69) | 4 (14) | 5 (17) |
|  | Mild (45-55) | 11 (37) | 13 (45) |  |  |  |
|  | Moderate (>55-60) | 12 (40) | 5 (17) |  |  |  |
|  | Severe (>60) | 6 (20) | 3 (10) |  |  |  |
| Anxiety ^b^ | | | | | | |
| LOW | Normal (<55) | 10 (33) | 16 (61) | 14 (54) | 8 (31) | 4 (16) |
|  | Mild (55-64) | 15 (50) | 8 (31) |  |  |  |
|  | Moderate (65-74) | 5 (17) | 2 (8) |  |  |  |
| HIGH | Normal (<55) | 11 (37) | 16 (55) | 16 (55) | 10 (34) | 3 (10) |
|  | Mild (55-64) | 16 (53) | 13 (45) |  |  |  |
|  | Moderate (65-74) | 3 (10) | 0 (0) |  |  |  |
| Depression ^b^ | | | | | | |
| LOW | Normal (<55) | 16 (53) | 20 (77) | 12 (46) | 11 (42) | 3 (12) |
|  | Mild (55-64) | 12 (40) | 6 (23) |  |  |  |
|  | Moderate (65-74) | 2 (7) | 0 (0) |  |  |  |
| HIGH | Normal (<55) | 19 (63.3) | 22 (76) | 18 (62) | 8 (28) | 3 (10) |
|  | Mild (55-64) | 7 (23.3) | 7 (24) |  |  |  |
|  | Moderate (65-74) | 4 (13.3) | 0 (0) |  |  |  |
| Satisfaction with social roles and activities ^e^ | | | | | | |
| LOW | Very high (>70) | 0 (0) | 0 (0) | 15 (58) | 7 (27) | 4 (15) |
|  | High (70-59) | 3 (10) | 2 (7) |  |  |  |
|  | Average (60-40) | 15 (50) | 21 (81) |  |  |  |
|  | Low (39-30) | 9 (30) | 3 (12) |  |  |  |
|  | Very low (<30) | 3 (10) | 0 (0) |  |  |  |
| HIGH | Very high (>70) | 0 (0) | 0 (0) | 22 (76) | 4 (14) | 3 (10) |
|  | High (70-59) | 1 (3) | 5 (17) |  |  |  |
|  | Average (60-40) | 21 (70) | 22 (76) |  |  |  |
|  | Low (39-30) | 6 (20) | 2 (7) |  |  |  |
|  | Very low (<30) | 2 (7) | 0 (0) |  |  |  |

Percentages do not always equal 100, due to rounding. ^a^ Loss to follow-up (LOW (n = 4), HIGH (n = 1)). Assessed using PROMIS Bank v1.2 Upper-Extremity and PROMIS-43 Profile v1.0. Cut-offs defined by ^b^ (35), ^c^ (36), ^d^ (42), ^e^ (37). Improved or declined = change in either direction ≥ 3 T-scores, maintained = change in either direction < 3 T-scores (37, 38); For pain NRS, improved or declined=change ≥2, maintained = change in either direction < 2 (36). The option “Severe” is not shown where no participants reported this severity level.

**Table S6**. Severity of self-reported upper-extremity function and breast cancer-relevant survivorship outcomes at baseline and 12 weeks, and proportion of improvers, decliners and maintainers over time in the LOW versus HIGH supervision exercise groups, in participants with lymphedema at baseline.^a^

| Group | PROMIS outcomes with severity benchmarks | Baseline  n% | 12 weeks  n% | Change in severity  from baseline (n (%)) | | |
| --- | --- | --- | --- | --- | --- | --- |
|  |  |  |  | Improve | Maintain | Decline |
| Upper-extremity function ^e^ | | | | | | |
| LOW | Normal (> 45) | 5 (50) | 5 (56) | 3 (33) | 6 (66) | 0 (0) |
|  | Mild dysfunction (40-45) | 2 (20) | 2 (22) |  |  |  |
|  | Moderate dysfunction (39-30) | 2 (20) | 2 (22) |  |  |  |
|  | Severe dysfunction (<30) | 1 (10) | 0 (0) |  |  |  |
| HIGH | Normal (> 45) | 2 (20) | 3 (30) | 4 (40) | 6 (60) | 0 (0) |
|  | Mild dysfunction (40-45) | 3 (30) | 4 (40) |  |  |  |
|  | Moderate dysfunction (39-30) | 5 (50) | 3 (30) |  |  |  |
| Fatigue ^b^ | | | | | | |
| LOW | Normal (<50) | 2 (20) | 2 (22) | 6 (67) | 2 (22) | 1 (11) |
|  | Mild (50–54) | 0 (0) | 3 (33) |  |  |  |
|  | Moderate (55–74) | 8 (80) | 4 (44) |  |  |  |
| HIGH | Normal (<50) | 0 (0) | 3 (30) | 7 (78) | 3 (3) | 0 (0) |
|  | Mild (50–54) | 0 (0) | 1 (10) |  |  |  |
|  | Moderate (55–74) | 10 (100) | 6 (60) |  |  |  |
| Pain interference ^b^ | | | | | | |
| LOW | Normal (<50) | 2 (20) | 3 (33) | 7 (78) | 1 (11) | 1 (11) |
|  | Mild (50–59) | 7 (70) | 5 (56) |  |  |  |
|  | Moderate (60–69) | 0 (0) | 1 (11) |  |  |  |
|  | Severe (≥70) | 1 (10) | 0 (0) |  |  |  |
| HIGH | Normal (<50) | 2 (20) | 4 (40) | 6 (60) | 4 (40) | 0 (0) |
|  | Mild (50–59) | 1 (10) | 4 (40) |  |  |  |
|  | Moderate (60–69) | 6 (60) | 2 (20) |  |  |  |
|  | Severe (≥70) | 1 (10) | 0 (0) |  |  |  |
| Pain NRS (0-10) ^c^ | | | | | | |
| LOW | None 0 | 2 (20) | 1 (11) | 1 (11) | 5 (56) | 3 (33) |
|  | Mild (1-3) | 4 (40) | 4 (44) |  |  |  |
|  | Moderate (4-6) | 4 (40) | 4 (44) |  |  |  |
| HIGH | None 0 | 1 (10) | 2 (20) | 2 (20) | 6 (60) | 2 (20) |
|  | Mild (1-3) | 4 (40) | 4 (40) |  |  |  |
|  | Moderate (4-6) | 3 (30) | 2 (20) |  |  |  |
|  | Severe ≥7 | 2 (20) | 2 (20) |  |  |  |
| Physical function ^d^ | | | | | | |
| LOW | Normal (>50) | 2 (20) | 2 (22) | 5 (56) | 4 (44) | 0 (0) |
|  | Mild dysfunction (35-50) | 7 (70) | 7 (78) |  |  |  |
|  | Moderate dysfunction(<35- 20) | 1 (10) | 0 (0) |  |  |  |
| HIGH | Normal (>50) | 0 (0) | 1 (10) | 5 (50) | 3 (30) | 2 (20) |
|  | Mild dysfunction (35-50) | 8 (80) | 7 (70) |  |  |  |
|  | Moderate dysfunction(<35- 20) | 2 (20) | 2 (20) |  |  |  |
| Sleep disturbance ^d^ | | | | | | |
| LOW | Normal (<45) | 0 (0) | 1 (11) | 5 (56) | 3 (33) | 1 (11) |
|  | Mild (45-55) | 4 (40) | 4 (44) |  |  |  |
|  | Moderate (>55-60) | 1 (10) | 3 (33) |  |  |  |
|  | Severe (>60) | 5 (50) | 1 (11) |  |  |  |
| HIGH | Normal (<45) | 0 (0) | 3 (30) | 7 (70) | 3 (30) | 0 (0) |
|  | Mild (45-55) | 3 (30) | 3 (30) |  |  |  |
|  | Moderate (>55-60) | 4 (40) | 2 (20) |  |  |  |
|  | Severe (>60) | 3 (30) | 2 (20) |  |  |  |
| Anxiety ^b^ | | | | | | |
| LOW | Normal (<55) | 3 (30) | 5 (56) | 4 (44) | 4 (44) | 1 (11) |
|  | Mild (55-64) | 6 (60) | 4 (44) |  |  |  |
|  | Moderate (65-74) | 1 (10) | 0 (0) |  |  |  |
| HIGH | Normal (<55) | 3 (30) | 4 (40) | 7 (70) | 3 (30) | 0 (0) |
|  | Mild (55-64) | 5 (50) | 6 (60) |  |  |  |
|  | Moderate (65-74) | 2 (20) | 0 (0) |  |  |  |
| Depression ^b^ | | | | | | |
| LOW | Normal (<55) | 4 (40) | 6 (67) | 5 (56) | 4 (44) | 0 (0) |
|  | Mild (55-64) | 5 (50) | 3 (33) |  |  |  |
|  | Moderate (65-74) | 1 (10) | 0 (0) |  |  |  |
| HIGH | Normal (<55) | 6 (60) | 6 (60) | 7 (70) | 2 (20) | 1 (10) |
|  | Mild (55-64) | 2 (20) | 4 (40) |  |  |  |
|  | Moderate (65-74) | 2 (20) | 0 (0) |  |  |  |
| Satisfaction with social roles and activities ^e^ | | | | | | |
| LOW | Very high (>70) | 0 (0) | 0 (0) | 5 (55) | 2 (22) | 2 (22) |
|  | High (70-59) | 2 (20) | 1 (11) |  |  |  |
|  | Average (60-40) | 4 (40) | 6 (67) |  |  |  |
|  | Low (39-30) | 2 (20) | 2 (22) |  |  |  |
|  | Very low (<30) | 2 (20) | 0 (0) |  |  |  |
| HIGH | Very high (>70) | 0 (0) | 0 (0) | 7 (70) | 2 (20) | 1 (10) |
|  | High (70-59) | 0 (0) | 2 (20) |  |  |  |
|  | Average (60-40) | 7 (70) | 7 (70) |  |  |  |
|  | Low (39-30) | 1 (10) | 1 (10) |  |  |  |
|  | Very low (<30) | 2 (20) | 0 (0) |  |  |  |
|  | Mild dysfunction (40-45) | 3 (30) | 4 (40) |  |  |  |
|  | Moderate dysfunction (39-30) | 5 (50) | 3 (30) |  |  |  |

^a^Lymphedema (L-Dex > 7.1 or bilateral criterion (as described in text) or self-reported clinical diagnosis). Percentages do not always equal 100, due to rounding. Assessed using PROMIS Bank v1.2 Upper-Extremity and PROMIS-43 Profile v1.0. Cut-offs defined by; ^b^ (35), ^c^ (43), ^d^ (42), ^e^ (37). Improved or declined = change ≥ 3 T-scores, maintained = change in either direction < 3 T-scores (37, 38); For pain NRS, improved or declined = change ≥2, maintained = change in either direction < 2 (36). The option “Severe” is not shown where no participants reported this severity level.
